# Supplementary material for: Spatiotemporal manipulation of ciliary glutamylation reveals its roles in intraciliary trafficking and Hedgehog signaling
Source: Nat Commun. 2018 Apr 30;9:1732. doi: 10.1038/s41467-018-03952-z (PMC5928066; doi:10.1038/s41467-018-03952-z)
Supplement: Supplementary file 1 — upplementary Information [file 41467_2018_3952_MOESM1_ESM.docx]

**Spatiotemporal manipulation of ciliary glutamylation reveals its roles in intraciliary trafficking and Hedgehog signaling**

**Hong et al.**

**
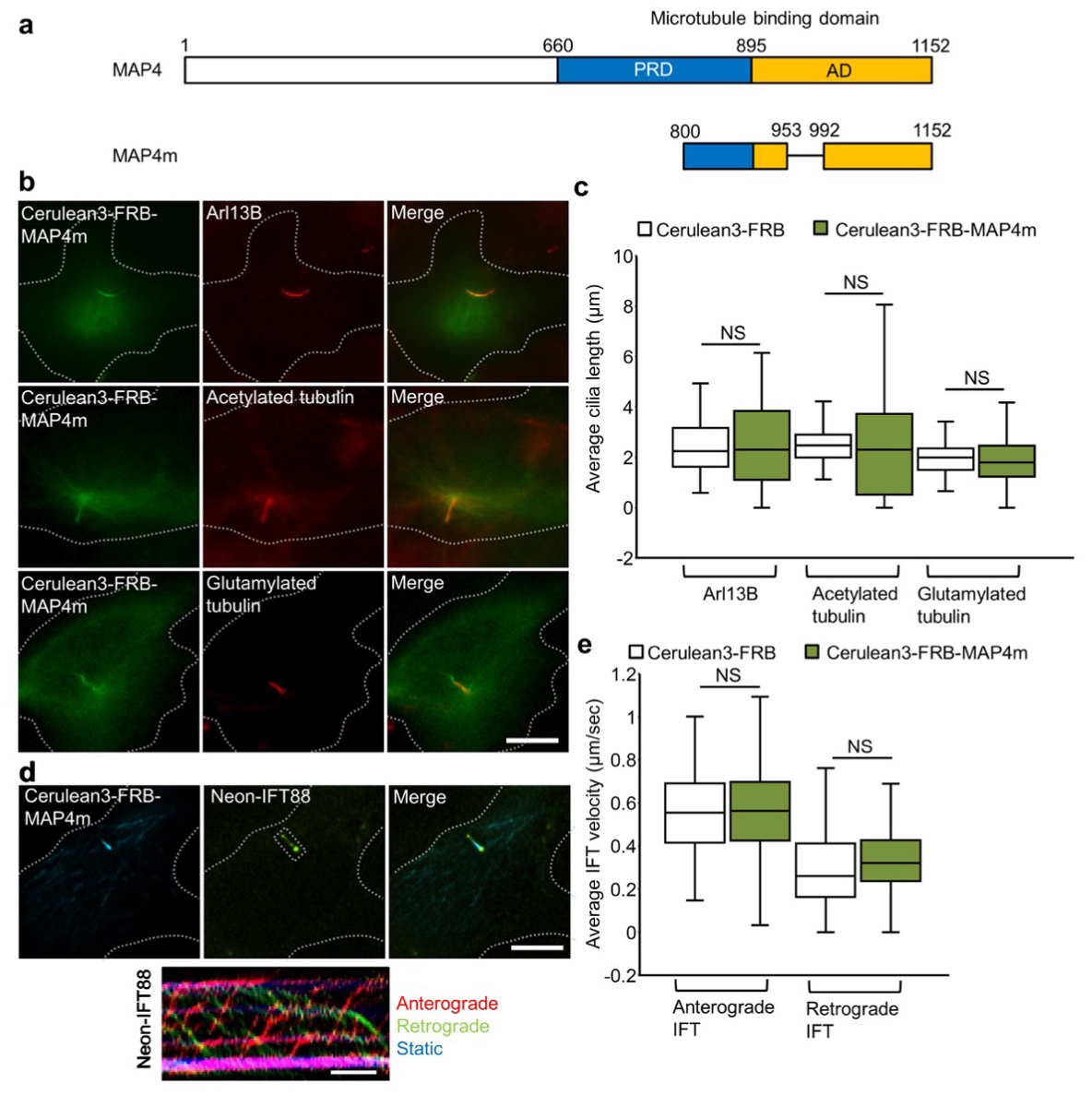
**

**Supplementary Fig. 1** MAP4m localizes at the ciliary axoneme without affecting the ciliary structure and intraflagellar transport. **a** Schematic diagram of MAP4 and truncated MAP4m protein. The proline-rich domain (PRD) and affinity domain (AD) in MAP4 protein are shown. **b** Cerulean3-FRB-MAP4m (green) localizes at the axoneme in NIH3T3 cells. NIH3T3 cells were transfected with Cerulean3-FRB-MAP4m. Transfected cells at 80~90% confluency were serum starved for 24 h and then stained by the indicated antibodies. Arl13B marks ciliary membrane (red). Acetylated tubulin and glutamylated tubulin mark the axoneme (red). Scale bars, 5 μm. **c** Expression of MAP4m does not affect cilium length. Box plots of cilium length measured using Arl13B, acetylated tubulin, glutamylated tubulin shown in **b**. (n = 69, 36, 36, 106, 35, 146 from left to right; three independent experiments) **d** Expression of MAP4m does not affect the dynamics of IFT. The Neon-IFT88 stable NIH3T3 cells were transfected with Cerulean3-FRB or Cerulean3-FRB-MAP4m. Transfected cells at 80~90% confluency were serum starved for 24 h and imaged by time-lapse imaging. The kymograph of IFT dynamics was created by KymographClear. Scale bar, 10 s. **e** The rates of IFT were tracked and quantified by the movement of Neon-IFT88 in anterograde and retrograde directions. (n = 105, 90, 100, 66 Neon-IFT88 particles from left to right; 3-5 independent experiments). NS represents no significant difference between the control group and the cells expressing Cereulan3-FRB-MAP4m (Student’s *t*-test).


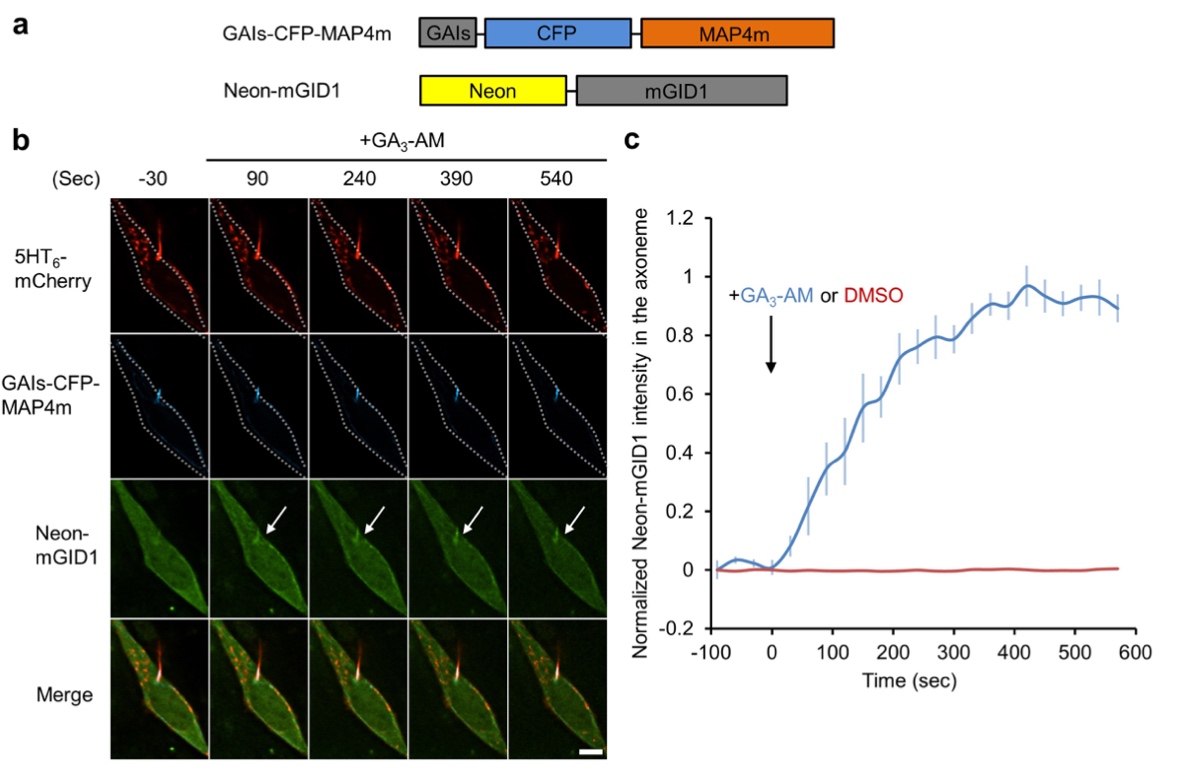


**Supplementary Fig. 2** Translocation of cytosolic proteins onto the axoneme using a gibberellin-induced dimerization system. **a** Schematic diagram of the constructs that encoded GAIs-CFP-MAP4m and Neon-mGID1 proteins. **b** The addition of 100 μM GA_3_-AM induces accumulation of Neon-mGID1 onto the GAIs-CFP-MAP4m-labeled axoneme (arrows). NIH3T3 cells were co-transfected with 5HT_6_-mCherry, GAIs-CFP-MAP4m and Neon-mGID1. Transfected cells at 80~90% confluency were serum starved for 24 h and then treated with 100 μM GA_3_-AM for the indicated time. 5HT_6_-mCherry marks ciliary membrane. Scale bar, 5 μm. Also, see Supplementary Movie 2. **c** Time course of Neon-mGID1 fluorescence intensity in the axoneme treated with 100 μM GA_3_-AM (blue) or 0.1% DMSO (red). (n = 6 cells in the GA_3_-AM treated group; n = 8 cells in the DMSO treated group; three independent experiments) Data represent the mean ± s.e.m.


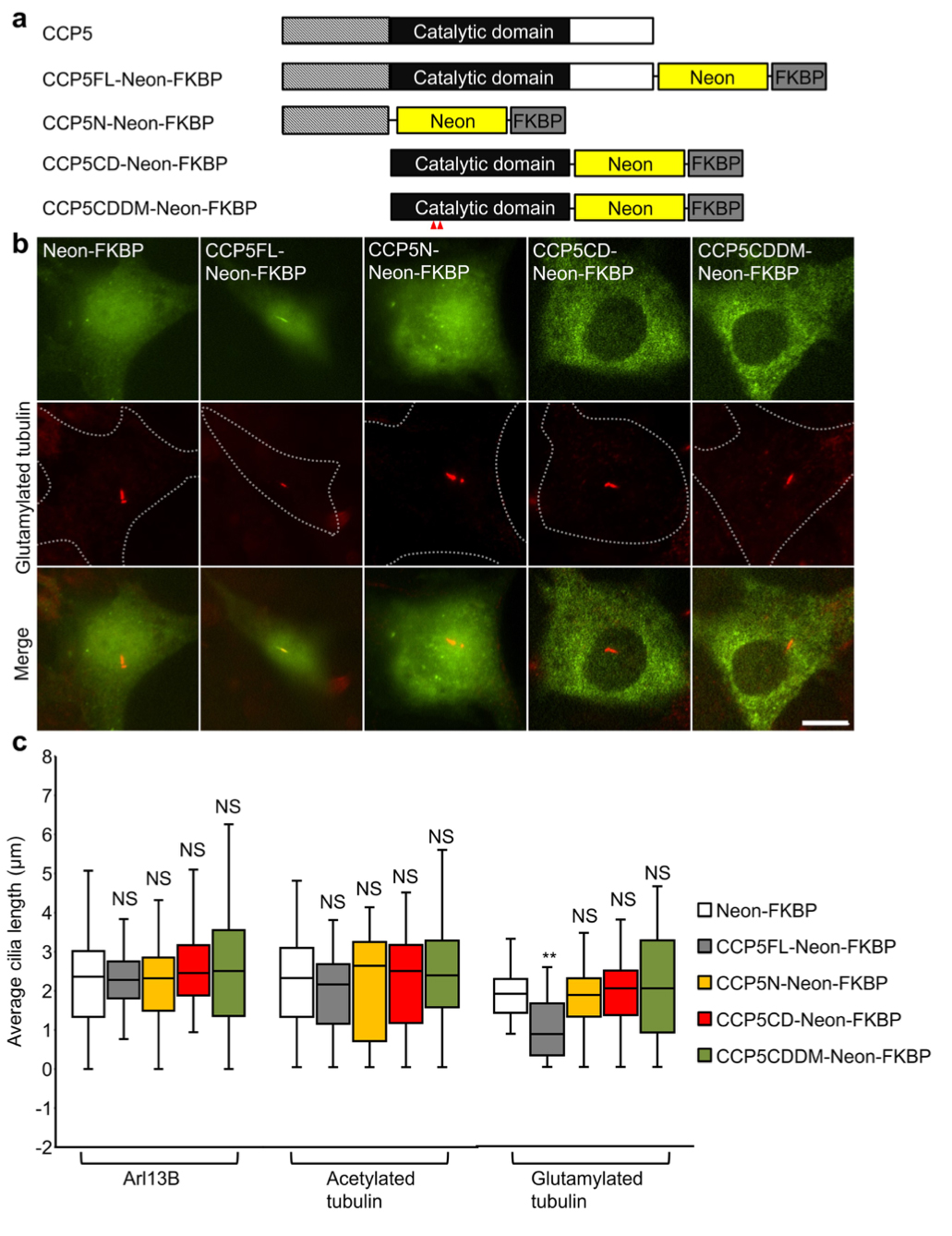


**Supplementary Fig. 3** Characterization of various domains in CCP5 protein. **a** Schematic diagram of Neon-FKBP tagged full length (CCP5FL-Neon-FKBP), N-terminus (CCP5N-Neon-FKBP), wild type catalytic domain (CCP5CD-Neon-FKBP), and catalytically inactive form of CCP5 (CCP5CDDM-Neon-FKBP). **b** CCP5FL-Neon-FKBP but not CCP5N-Neon-FKBP, CCP5CD-Neon-FKBP, or CCP5CDDM-Neon-FKBP (green) localizes in cilia and reduces the axonemal glutamylation. NIH3T3 cells were transfected with the indicated Neon-FKBP-tagged proteins. Transfected cells at 80~90% confluency were serum starved for 24 h, followed by stained with glutamylated tubulin (red). Scale bar, 10 μm. **c** CCP5FL-Neon-FKBP but not other CCP5 domain significantly reduces the axonemal glutamylation without affecting the ciliary length and axonemal acetylation. Box plots of cilia length labeled by antibodies against Arl13B, acetylated tubulin, and glutamylated tubulin, respectively, are shown. (n = 67, 30, 31, 45, 30, 44, 30, 33, 31, 47, 50, 47, 36, 33, 34 cells from left to right; 3-5 independent experiments). NS and ** represent no significant difference and *P* < 0.01 between the control (Neon-FKBP) and the cells expressing indicated proteins (Student’s *t*-test).


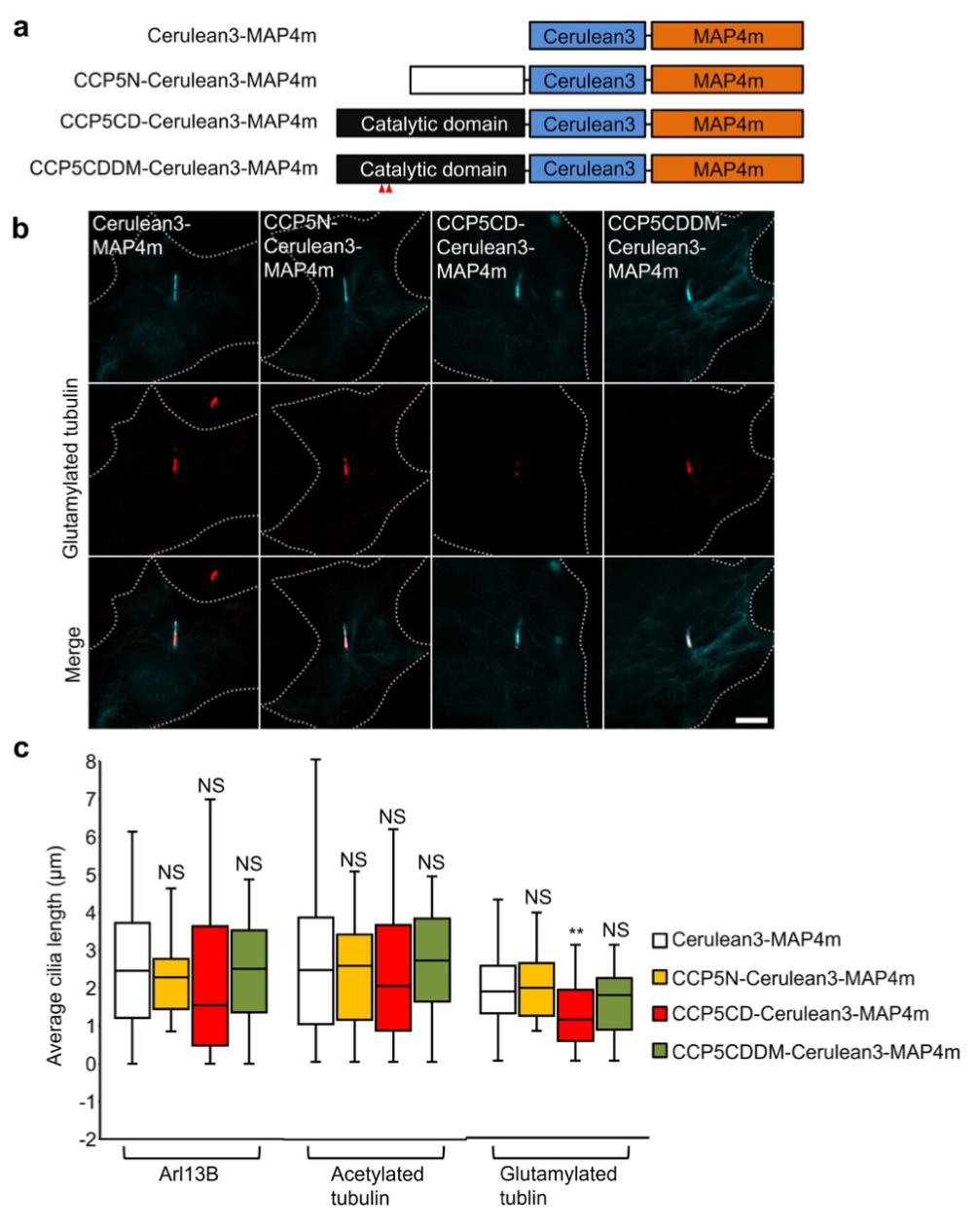


**Supplementary Fig. 4** Targeting CCP5CD to the axoneme reduces axonemal glutamylation. **a** Schematic diagram of the various Cerulean3-MAP4m-fused CCP5N, CCP5CD, and CCP5CDDM. **b** CCP5CD-Cerulean3-MAP4m localizes in the axoneme and reduces axonemal glutamylation. NIH3T3 cells were transfected with indicated Cerulean3-MAP4m-tagged proteins. Transfected cells at 80~90% confluency were serum starved for 24 h and stained for glutamylated tubulin (red). Scale bar, 5 μm. **c** CCP5CD-Cerulean3-MAP4m but not other CCP5 constructs significantly reduces the axonemal glutamylation without affecting ciliary length and axonemal acetylation. Box plots of cilia length labeled by antibodies against Arl13B, acetylated tubulin, and glutamylated tubulin, respectively, are shown. (n = 94, 50, 30, 36, 146, 60, 49, 30, 127, 31, 41, 34 cells from left to right; four independent experiments). NS and ** represent no significant difference and *P* < 0.01 between the control (Cerulean3-MAP4m) and the cells expressing indicated proteins (Student’s *t*-test).


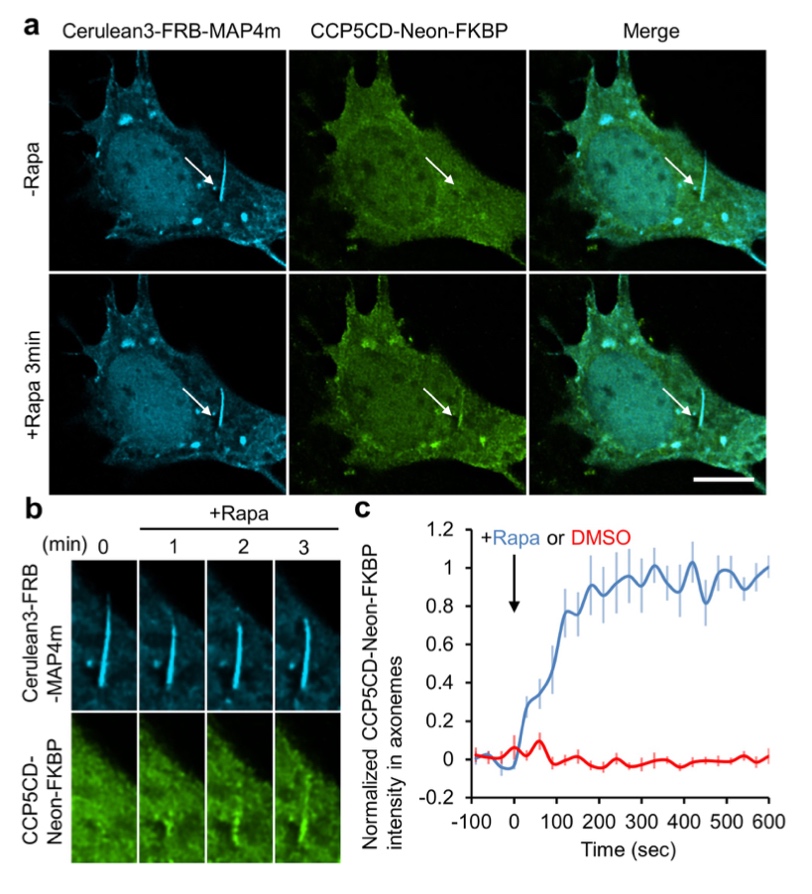


**Supplementary Fig. 5** Translocation of CCP5CD-Neon-FKBP onto the ciliary axoneme using a rapamycin dimerization system. **a** The addition of 100 nM rapamycin induces accumulation of CCP5CD-Neon-FKBP onto the Cerulean3-FRB-MAP4m-labeled axoneme (arrows). NIH3T3 cells were co-transfected with Cerulean3-FRB-MAP4m and CCP5CD-Neon-FKBP. Transfected cells at 80~90% confluency were serum starved for 24 h and treated with 100 nM rapamycin. Scale bar, 5 μm. Also, see Supplementary Movie 3. **b** Individual video frames in the axoneme region of cell in **a** upon 100 nM rapamycin treatment. **c** Time course of YFP fluorescence intensity in the axoneme of NIH3T3 cells treated with 100 nM rapamycin (blue) or 0.1% DMSO (red). Data represent the mean ± s.e.m. (n = 11 cells for the DMSO group, n = 7 cells for the rapamycin group; three independent experiments).

**
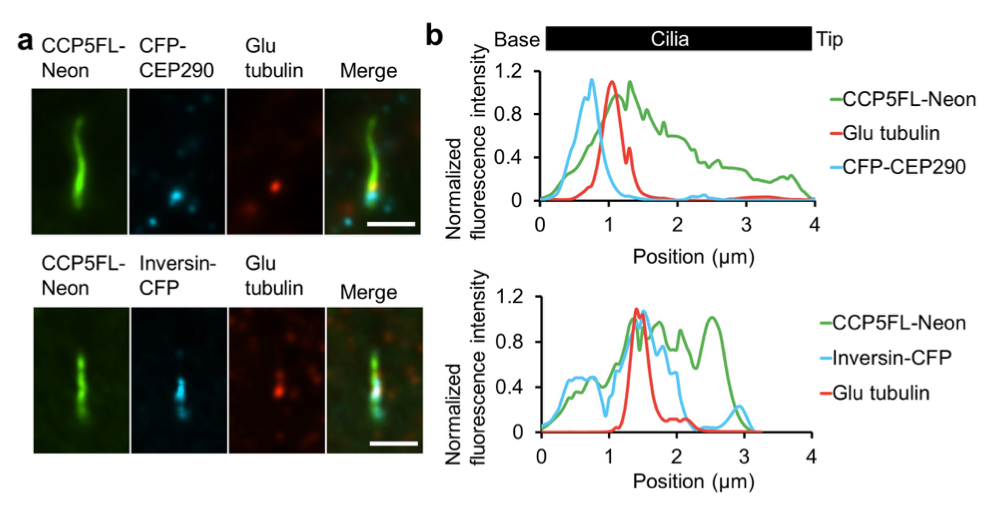
**

**Supplementary Fig. 6** The distribution of residual glutamylated tubulin after axonemal deglutamylation. **a** Residual glutamylated tubulin localize at the inversin zone but not the transition zone after deglutamylation. NIH3T3 were transfected with CCP5FL-Neon and CFP-CEP290 or Inversin-CFP constructs for 24 h. Transfected cells at 80~90% confluency were serum starved for 24 h and then stained with anti-Glutamylated tubulin antibody. Scale bar, 2 μm. **b** Linescan profiles of the indicated proteins from the base to the tip of cilia in **a**.

**
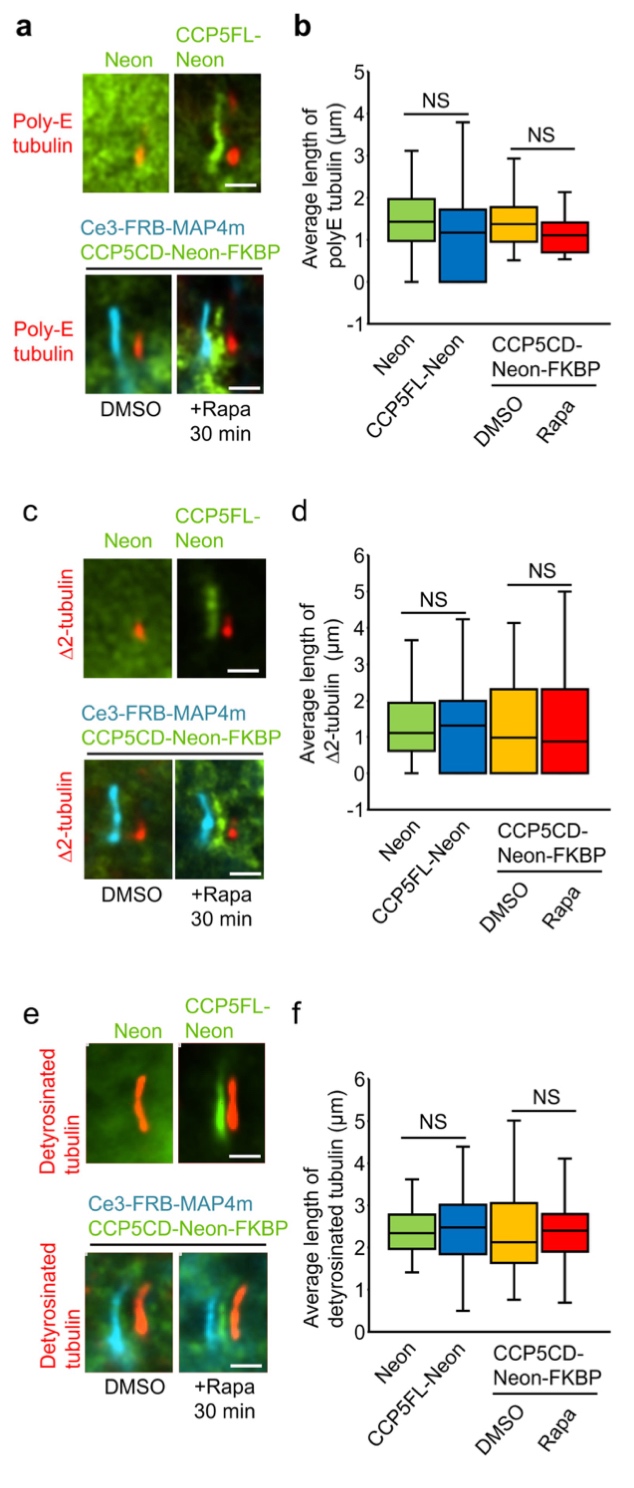
**

**Supplementary Fig. 7** The enzyme activity of STRIP in poly-glutamate side chain shortening, ∆2-∆3 tubulin conversion, and tubulin detyrosination. **a,b** CCP5 in cilia does not affect axonemal poly-glutamate (poly-E) **a**,**b**, ∆2-tubulin **c**.**d**, or axonemal detyrosination **e, f**. NIH3T3 cells were transfected with Neon, CCP5FL-Neon, or Cerulean3(Ce3)-FRB-MAP4m-P2A-CCP5CD-Neon-FKBP. Transfected cells at 80~90% confluency were serum starved for 24 h, followed by a treatment of 0.1% DMSO or 100 nM rapamycin for 30 min. The level of ciliary poly-glutamate side chain, ∆2-tubulin, and tubulin detyrosination in cells expressing the indicated proteins upon 0.1% DMSO or 100 nM rapamycin treatment (Rapa) for 30 min was assessed by labeling with specific antibodies and were quantified. The images show shifted overlays of indicated proteins in cilia. Scale bar, 2 μm. (n = 169, 121, and 291 cells in the poly-E, ∆2-tubulin, and tubulin detyrosination experiments, respectively; three independent experiments). NS indicates no significant difference between the control and the indicated groups (Student’s *t*-test).

**
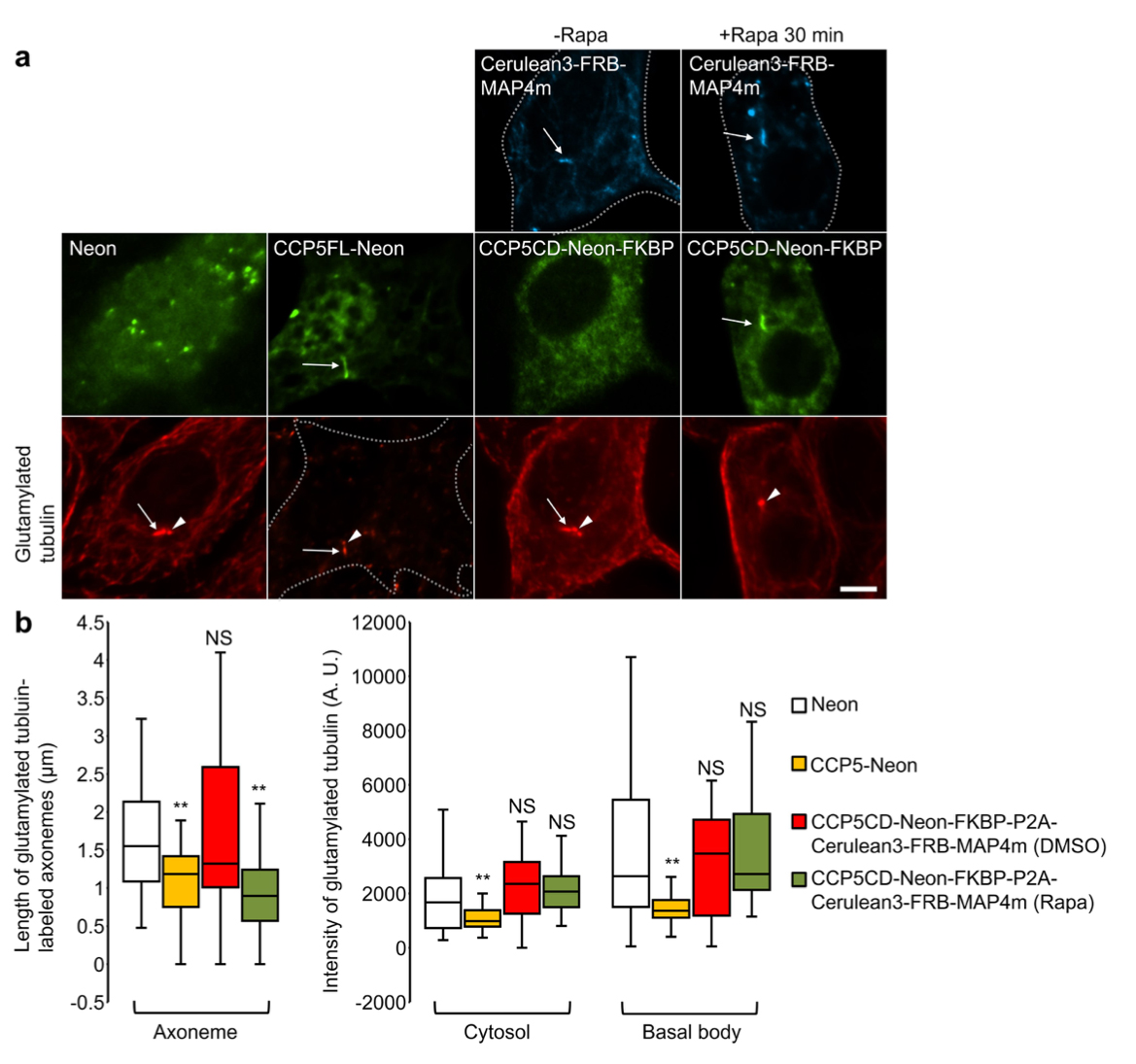
**

**Supplementary Fig. 8** Specifically depleting axonemal glutamylation by our STRIP system. **a** CCP5FL-Neon but not CCP5CD-Neon-FKBP globally depletes glutamylation in NIH3T3 cells. Translocation of CCP5CD-Neon-FKBP onto the Cerulean3-FRB-MAP4m-labeled axoneme specifically strips the axonemal glutamylation without affecting the tubulin glutamylation in the cytosol and basal body. NIH3T3 cells were transfected with Neon, CCP5FL-Neon, or Cerulean3-FRB-MAP4m-P2A-CCP5CD-Neon-FKBP. Transfected cells at 80~90% confluency were serum starved for 24 h and then treated with 0.1% DMSO (-Rapa) or 100 nM rapamycin (+Rapa) for 30 min. Subsequently, cells were incubated on ice for 1 h and stained with anti-glutamylated tubulin. Arrows and arrowheads mark the axoneme and basal body, respectively. Scale bars, 5 μm. **b** The length of glutamylated axoneme and the level of tubulin glutamylation in the cytosol and basal body in various conditions. (n = 71, 18, 25, 24, 206, 18, 34, 31, 197, 18, 29, 27 cells from left to right; 3-5 independent experiments). NS and ** represent no significant difference and *P* < 0.01, respectively, between the control (Neon) and the indicated conditions (Student’s *t*-test).


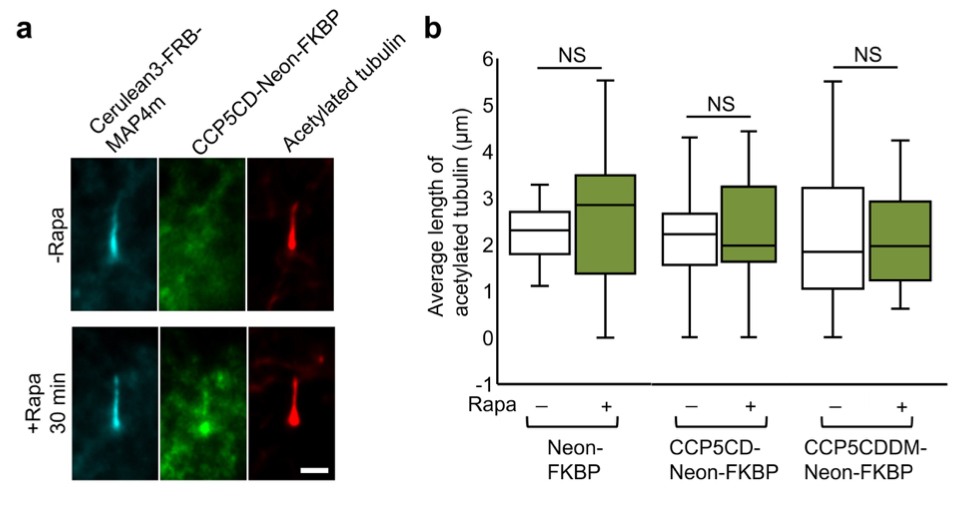


**Supplementary Fig. 9** Rapid deglutamylation does not affect axonemal acetylation. **a** Translocation of CCP5CD onto the axoneme does not affect the level of acetylated tubulin. NIH3T3 cells were transfected with P2A-based constructs for co-expression of Cerulean3-FRB-MAP4m and Neon-FKBP-tagged proteins. Transfected cells at 80~90% confluency were serum starved for 24 h and treated with or without 100 nM rapamycin for 30 min. Subsequently, cells were stained with anti-acetylated tubulin antibody. Scale bar, 2 μm. **b** Average length of acetylated tubulin in cells expressing the indicated proteins in the presence or absence of 100 nM rapamycin treatment for 30 min were quantified. (n = 13, 19, 20, 17, 19, 15 cells from left to right; three independent experiments). NS represents no significant difference between groups with or without rapamycin treatment (Student’s *t*-test).

**
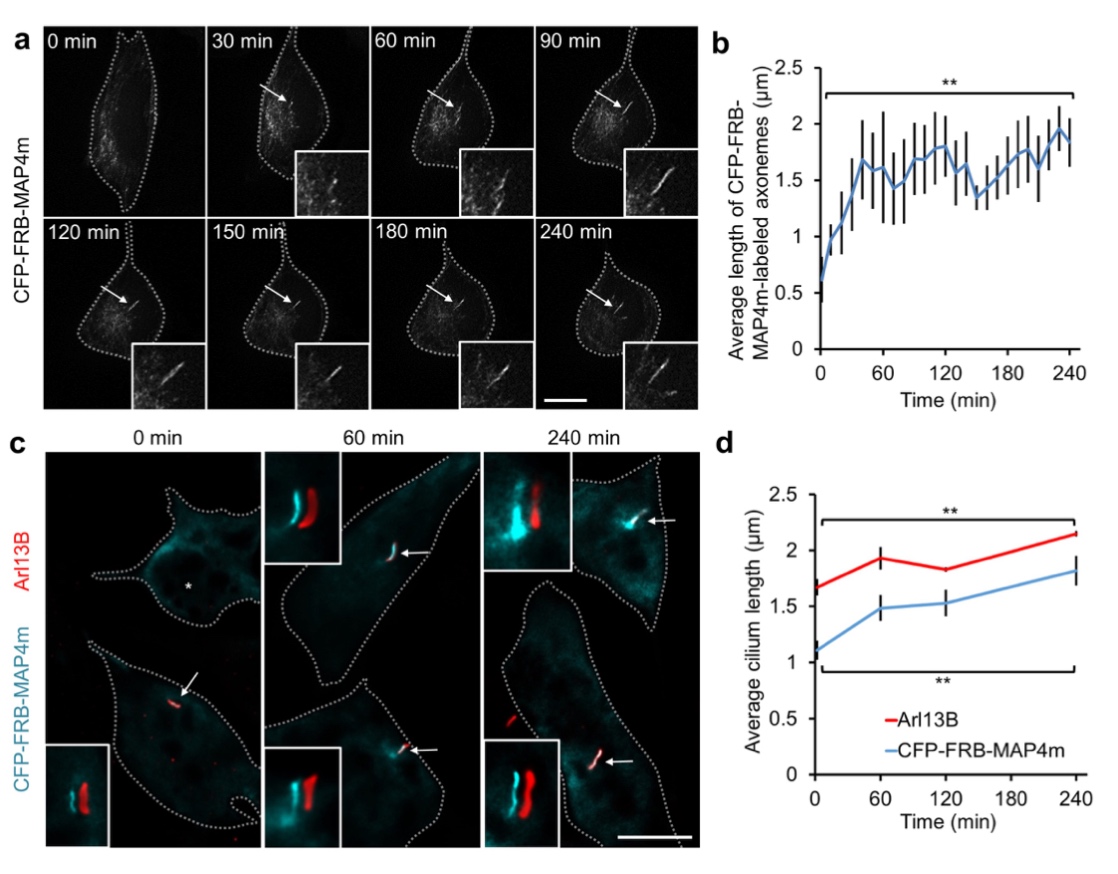
**

**Supplementary Fig. 10** MAP4m serves as an axoneme targeting protein during ciliogenesis. **a** Time-lapse imaging of a NIH3T3 cell stably expressing CFP-FRB-MAP4m after serum starvation for the indicated times. Magnified views of the growing MAP4m-labeled axoneme (arrows) are shown in the insets. Scale bar, 10 μm. Also see Supplementary Movie 5. **b** Average length of the MAP4m-labeled axoneme after serum starvation for the indicated times was quantified. (n = 8 cells; two independent experiments). **c** The cilia in NIH3T3 cells stably expressing CFP-FRB-MAP4m (green) after serum starvation for the indicated times were stained with anti-Arl13B antibody (red). The insets show shifted overlays of MAP4m and Arl13B (arrows). A non-ciliated cell is marked by an asterisk. Scale bar, 10 μm. **d** Average length of the MAP4m-labeled axoneme and the Arl13B-labeled ciliary membrane after serum starvation for indicated times was quantified. (n≥ 180 cells; three independent experiments). Data represent the mean ± s.e.m. ** represent *P* < 0.01 between the cilium length before and after serum starvation for 240 min (Student’s *t*-test).


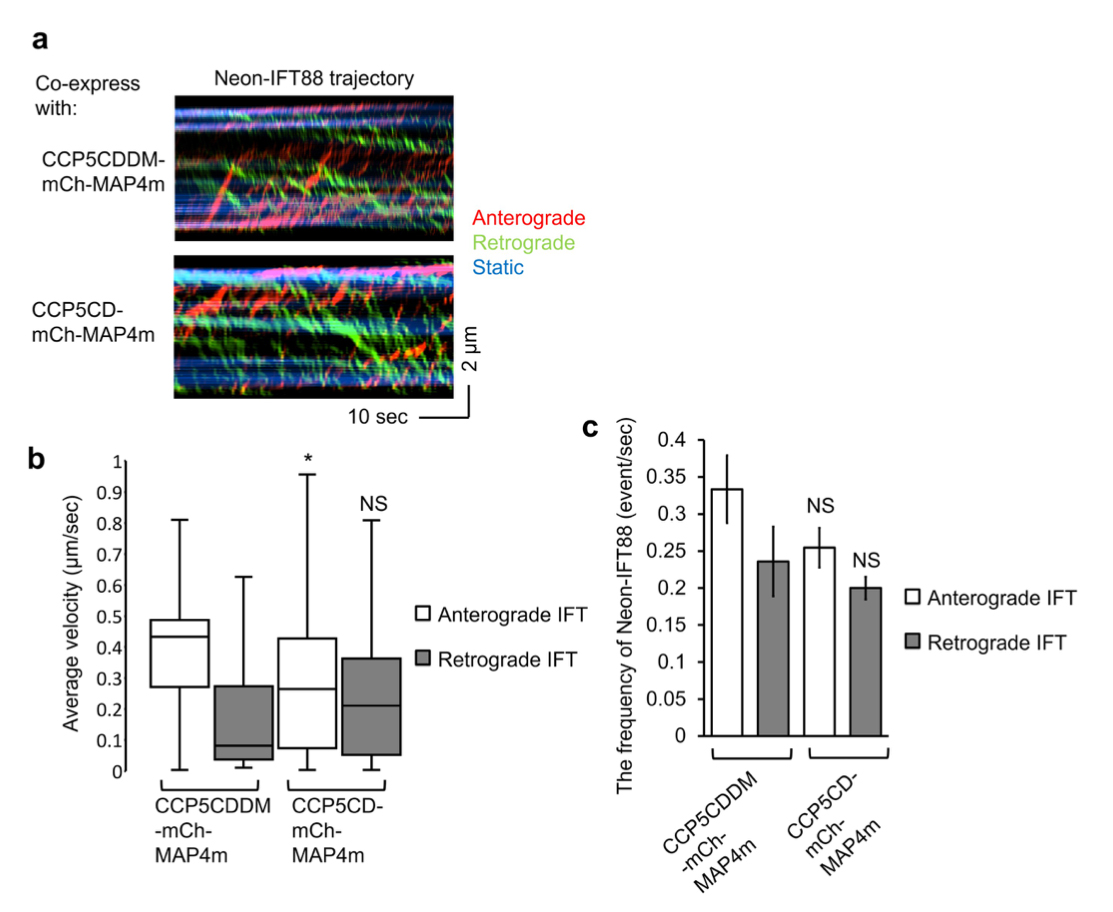


**Supplementary Fig. 11** Long-term deglutamylation preferentially hampers anterograde IFT. **a** The Neon-IFT88 stable NIH3T3 cells were transfected with CCP5CD-mCherry-MAP4m or catalytically inactive CCP5CDDM-mCherry-MAP4m. Transfected cells at 80~90% confluency were serum starved for 24 h. Representative kymographs of Neon-IFT88 in cells co-expressing CCP5CD-mCherry-MAP4m or CCP5CDDM-mCherry-MAP4m were generated from time-lapse imaging together with KymographClear. Red, green, and blue lines represent the trajectories of Neon-IFT88 particles in anterograde, retrograde directions and static Neon-IFT88, respectively. **b** The velocity of Neon-IFT88 in anterograde and retrograde direction was quantified according to the trajectories shown in kymographs (See the method). (n = 353, and 261 neon-IFT88 particles in the CCP5CD-mCherry-MAP4m and CCP5CDDM-mCherry-MAP4m groups, respectively; three independent experiments). **c** The frequency of Neon-IFT88 particles moving along cilia expressing CCP5CD-mCherry-MAP4m or CCP5CDDM-mCherry-MAP4m (n = 13 and 11 cilia in the CCP5CD-mCherry-MAP4m and CCP5CDDM-mCherry-MAP4m groups, respectively; three independent experiments). Data represent the mean ± s.e.m. NS and * indicate no significant difference and *P* < 0.05, respectively, between the CCP5CDDM-mCherry-MAP4m and the CCP5CD-mCherry-MAP4m groups (Student’s *t*-test).

**
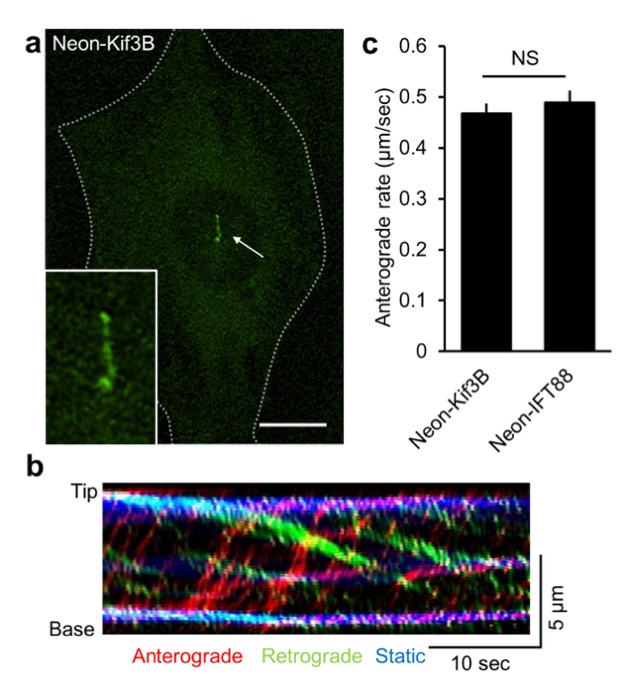
**

**Supplementary Fig. 12** Neon-Kif3B localizes and moves along the axoneme. **a** The distribution of Neon-Kif3B in NIH3T3 cells are shown. Magnified view of the cilium expressing Neon-Kif3B (arrow) is shown in the inset. Scale bar, 10 μm. **b** A kymograph was generated from time-lapse imaging of cilium expressing Neon-Kif3B. The trajectories of Neon-Kif3B particles in anterograde and retrograde direction are highlighted by red and green lines, respectively. **c** The velocity of Neon-Kif3B and Neon-IFT88 in anterograde direction. (n = 258, 158 IFT particles from left to right; four independent experiments. Data represent the mean ± s.e.m. NS represents no significant difference between the Neon-Kif3B and the Neon-IFT88 (Student’s *t*-test).


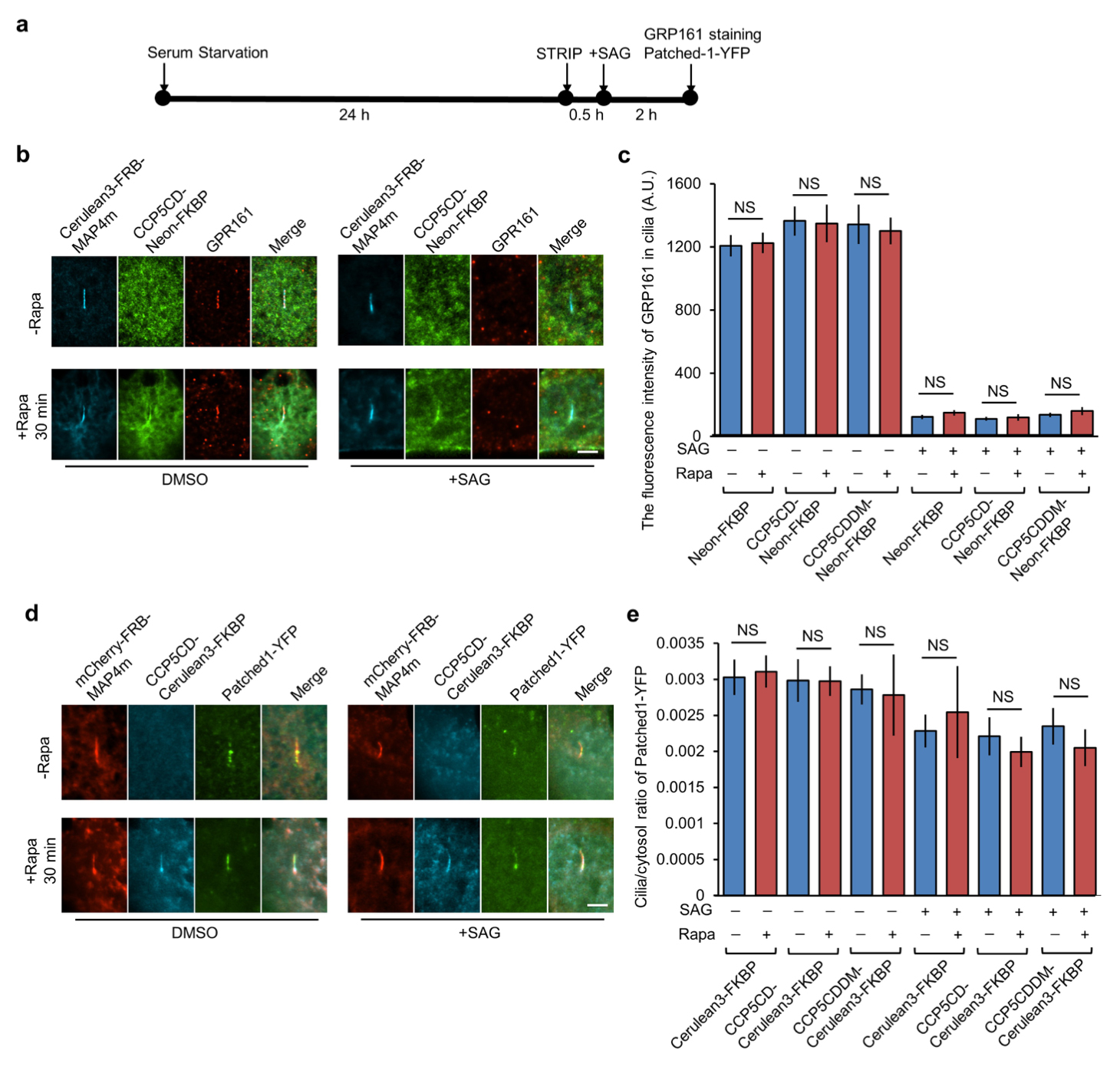


**Supplementary Fig. 13** Axonemal deglutamylation does not affect the ciliary exit of GPR161 and Patched1. **a** The experimental procedure for STRIP and Hedgehog induction. Washout of rapamycin after 30 min STRIP operation did not affect protein dimerization in cells owing to the irreversible nature of the CID system. **b** STRIP-induced axonemal deglutamylation does not affect the ciliary exit of GPR161 upon stimulation of the SAG. NIH3T3 cells were transfected with P2A-based constructs for co-expression of Cerulean3-FRB-MAP4m and Neon-FKBP-tagged proteins. Transfected cells at 80~90% confluency were serum starved for 24 h, followed by a treatment of 0.1% DMSO (-Rapa) or 100 nM rapamycin (+Rapa) for 30 min. Subsequently, cells were treated with 1 μM SAG for 2 h and stained with anti-GRP161 antibody. Scale bar, 2 μm. **c** The fluorescence intensity of GRP161 in cilia was quantified and plotted (n = 318, 235, and 218 cilia in the Neon-FKBP, CCP5CD-Neon-FKBP, and CCP5CDDM-Neon-FKBP groups, respectively; 3-5 independent experiments). Data represent the mean ± s.e.m. NS represents no significant difference between with and without rapamycin treatment group. **d** STRIP-induced axonemal deglutamylation does not affect the ciliary exit of Patched1-YFP upon stimulation of the SAG. NIH3T3 cells were transfected with Patched1-YFP and P2A-based constructs for co-expression of mCherry-FRB-MAP4m and Cerulean3-FKBP-tagged proteins. Transfected cells at 80~90% confluency were serum starved for 24 h and then treated with 0.1% DMSO (-Rapa) or 100 nM rapamycin (+Rapa) for 30 min. Subsequently, cells were treated with 1 μM SAG for 2 h. Scale bar, 2 μm. (**e**) The ratio of Patched1-YFP intensity in cilia and cytosol was quantified and plotted. (n = 201, 210, and 132 cilia in the Cerulean3-FKBP, CCP5CD-Cerulean3-FKBP, and CCP5CDDM-Cerulean3-FKBP groups, respectively; three independent experiments). NS represents no significant difference between the conditions in the presence and absence of rapamycin (Student’s *t*-test).


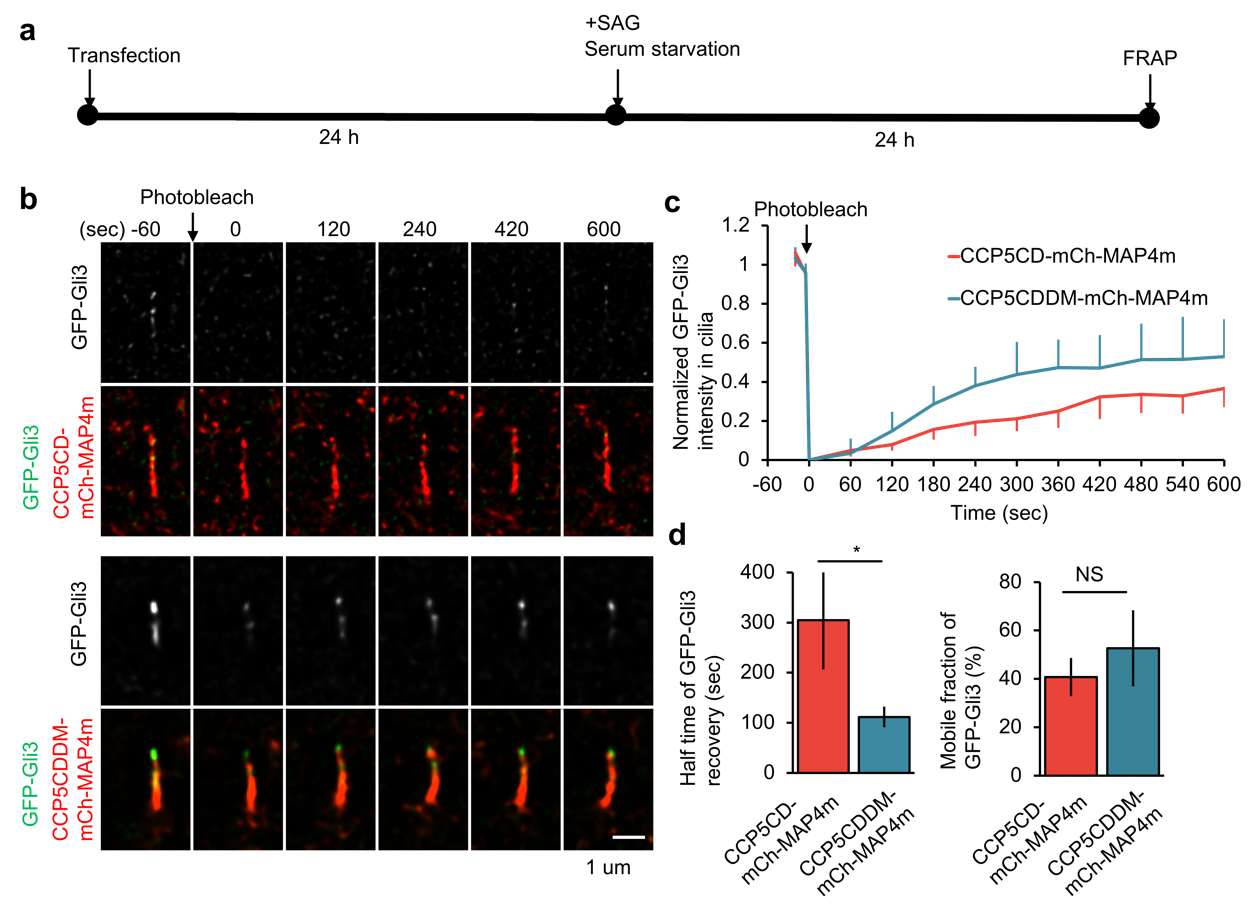


**Supplementary Fig. 14** Axonemal deglutamylation hampers ciliary entry of Gli3 upon SAG stimulation. **a** The experimental procedures in **b**. NIH3T3 cells were transfected with GFP-Gli3 and CCP5CD-mCherry-MAP4m or CCP5CDDM-mCherry-MAP4m for 24 h. Transfected cells at 80~90% confluency were treated with 200 nM SAG in serum free medium for 24 h and then analyzed by FRAP. **b** The GFP-Gli3 in cells expressing CCP5CD-mCherry-MAP4m or catalytically inactive CCP5CDDM-mCherry-MAP4m was photobleached and allowed for recovery for 10 min. Scale bar, 1 μm. **c** The fluorescence recovery of GFP-Gli3 in cilia expressing CCP5CD-mCherry-MAP4m or CCP5CDDM-mCherry-MAP4m was measured and plotted. **d** The recovery rate (left) and mobile fraction (right) of GFP-Gli3 in cilia were measured and plotted. Data represent the mean ± s.e.m. (n = 5, and 8 cilia for the CCP5CD-mCherry-MAP4m and CCP5CDDM-mCherry-MAP4m groups, respectively; three independent experiments). NS and * represent no significant difference and *P* < 0.05, respectively, between the CCP5CDDM-mCherry-MAP4m and the CCP5CD-mCherry-MAP4m groups (Student’s *t*-test).


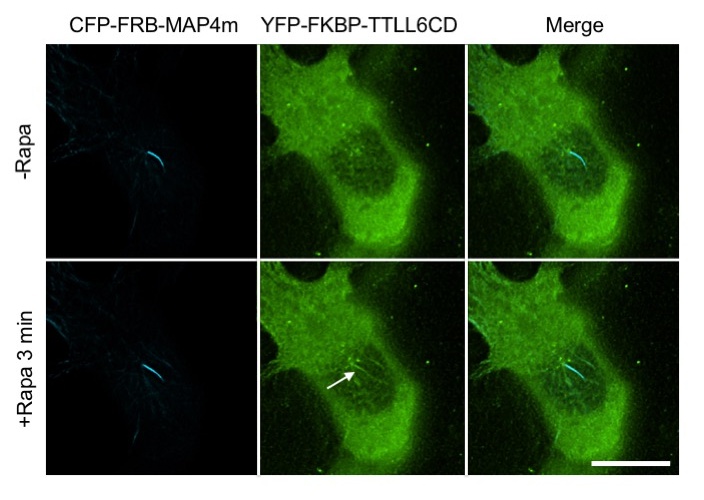


**Supplementary Fig. 15** Recruiting the catalytic domain TTLL6 onto ciliary axoneme. NIH3T3 cells were co-transfected with CFP-FRB-MAP4m and YFP-FKBP-TTLL6CD (the catalytic domain of TTLL6). Addition of rapamycin (100 nM) induces translocations of YFP-FKBP-TTLL6CD from cytosol to CFP-FRB-MAP4m-labeled axoneme (arrow head). Scale bar, 10 μm.
