# Supplementary material for: Spatiotemporal manipulation of ciliary glutamylation reveals its roles in intraciliary trafficking and Hedgehog signaling
Source: Nat Commun. 2018 Apr 30;9:1732. doi: 10.1038/s41467-018-03952-z (PMC5928066; doi:10.1038/s41467-018-03952-z)
Supplement: Supplementary file 3 — Description of Additional Supplementary Files [file 41467_2018_3952_MOESM3_ESM.pdf]

.

Supplementary Movie 1. Rapid translocation of cytosolic proteins onto the MAP4m-labeled ciliary axoneme.

NIH3T3 cells were co-transfected with YFP-FKBP and Cerulean3-FRB-MAP4m. Transfected cells at 80~90% confluency were serum starved for 24 h. The addition of 100 nM rapamycin leads to a rapid increase in YFP-FKBP fluorescence in the axoneme. Images were taken every 30 seconds for 19.5 min. Scale bar, 10 µm.

Supplementary Movie 2. Recruitment of cytosolic Neon-mGID1 onto the GAIs-CFP-MAP4m-labeled axoneme by gibberellin-dependent CID system.

NIH3T3 cells were co-transfected with $5HT_6$-mCherry, Neon-mGID1, GAIs-CFP-MAP4m. Transfected cells at 80~90% confluency were serum starved for 24 h. Addition of 100 µM GA_3-AM leads to a rapid increase in Neon-mGID1 fluorescence in the axoneme. Images were taken every 30 second for 10.5 min. Scale bar, 5 µm.

Supplementary Movie 3. Rapid translocation of CCP5CD-Neon-FKBP onto the MAP4m-labeled axoneme.

NIH3T3 cells were transfected with P2A-based constructs for co-expression of Cerulean3-FRB-MAP4m and CCP5CD-Neon-FKBPYFP-FKBP. Transfected cells at 80~90% confluency were serum starved for 24 h. Addition of 100 nM rapamycin leads to a rapid increase in CCP5CD-Neon-FKBP fluorescence in the axoneme. Arrows mark ciliary axoneme region. Images were taken every 30 seconds for 12.5 min. Scale bar, 10 µm.

Supplementary Movie 4. Deglutamylation induced by recruiting CCP5CD onto the axoneme does not affect the length and morphology of cilia.

NIH3T3 cells were co-transfected with $5HT_6$-mCherry, CCP5CD-Neon-FKBP, and Cerulean3-FRB-MAP4m. Transfected cells at 80~90% confluency were serum starved for 24 h. Addition of 100 nM rapamycin induces the translocation of CCP5CD from cytosol to the axoneme for local deglutamylation. The real-time morphology of primary cilium during deglutamylation was visualized by a ciliary membrane marker, $5HT_6$-mCherry. Images were taken every 2 min for 80 min. Scale bar, 2.5 µm.

Supplementary Movie 5. Real-time morphology of the MAP4m-labeled axoneme during ciliogenesis.

NIH3T3 cells stably expressing CFP-FRB-MAP4m were incubated with serum free medium,

and real-time morphology of the MAP4m-labeled axoneme was visualized by time-lapse imaging. Z-stack images of the cells with 0.5 µm Z-interval were taken every 10 min for 4 h. Left and right images showed the representing cells with no visible axoneme or short axoneme, respectively, in the beginning of serum starvation. Scale bar, 10 µm.

Supplementary Movie 6. The effect of axonemal deglutamylation on the dynamic of IFT.
The Neon-IFT88 stable NIH3T3 cells were co-transfected with mCherry-FRB-MAP4m and CCP5CD-cerulean3-FKBP. The dynamic of Neon-IFT88 in a cilium before and after CID-induced deglutamylation was visualized by time-lapse imaging. Images were taken every 200 millisecond for 15 seconds. Scale bar, 4 µm.

Supplementary Movie 7. Axonemal deglutamylation hampers the motility of IFT along axoneme but not the tethering of IFT on basal body.
The Neon-IFT88 stable NIH3T3 cells were transfected with CCP5CD-mCherry-MAP4m or catalytically inactive CCP5CDDM-mCherry-MAP4m. Transfected cells at 80~90% confluency were starved for 24 h prior to the FRAP experiments. The cell expressing indicated proteins were photobleached in the entire cilia region and allowed for recovery for 20 min. Images were taken every 60 second for 22 min. Scale bar = 2 µm.

Supplementary Movie 8. The motility of Neon-Kif3B.
The Neon-Kif3B stable NIH3T3 cells with 80~90% confluence were serum starved for 24 h and the dynamics of Neon-Kif3B was visualized by time-lapse imaging. Images were taken every 200 millisecond for 30 seconds. Scale bar, 5 µm.

Supplementary Movie 9. Axonemal deglutamylation hampers ciliary entry of Gli3 upon SAG stimulation.
NIH3T3 cells were transfected with GFP-Gli3 and CCP5CD-mCherry-MAP4m or CCP5CDDM-mCherry-MAP4m for 24 h. Transfected cells at 80~90% confluency were treated with 200 nM SAG in serum free medium for 24 h and analyzed by FRAP. The GFP-Gli3 in cilia region was photobleached and allowed for recovery for 20 min. Images were taken every 60 second for 22 min. Scale bar, 1 µm.
